# Head-to-Head Comparison of Three Vaccination Strategies Based on DNA and Raw Insect-Derived Recombinant Proteins against *Leishmania*

**DOI:** 10.1371/journal.pone.0051181

**Published:** 2012-12-07

**Authors:** Felicitat Todolí, Alhelí Rodríguez-Cortés, María del Carmen Núñez, Márcia D. Laurenti, Silvia Gómez-Sebastián, Fernando Rodríguez, Eva Pérez-Martín, José M. Escribano, Jordi Alberola

**Affiliations:** 1 LeishLAB–Servei d’Anàlisi de Fàrmacs, Departament de Farmacologia, de Terapèutica i de Toxicologia, Edifici V, Universitat Autònoma de Barcelona, Bellaterra, Barcelona, Spain; 2 Alternative Gene Expression S.L., Centro Empresarial, Parque Científico y Tecnológico de la Universidad Politécnica de Madrid, Pozuelo de Alarcón, Madrid, Spain; 3 Laboratorio Patologia de Moléstias Infecciosas, Faculdade de Medicina da Universidade de São Paulo, São Paulo, Brazil; 4 Centre de Recerca en Sanitat Animal (CReSA), UAB-IRTA, Campus de la UAB, Bellaterra, Barcelona, Spain; 5 Departamento de Biotecnología, Instituto Nacional de Investigación y Tecnología Agraria y Alimentaria, Madrid, Spain; University of Delhi, India

## Abstract

Parasitic diseases plague billions of people among the poorest, killing millions annually, and causing additional millions of disability-adjusted life years lost. Leishmaniases affect more than 12 million people, with over 350 million people at risk. There is an urgent need for efficacious and cheap vaccines and treatments against visceral leishmaniasis (VL), its most severe form. Several vaccination strategies have been proposed but to date no head-to-head comparison was undertaken to assess which is the best in a clinical model of the disease. We simultaneously assayed three vaccination strategies against VL in the hamster model, using KMPII, TRYP, LACK, and PAPLE22 vaccine candidate antigens. Four groups of hamsters were immunized using the following approaches: 1) raw extracts of baculovirus-infected *Trichoplusia ni* larvae expressing individually one of the four recombinant proteins (PROT); 2) naked pVAX1 plasmids carrying the four genes individually (DNA); 3) a heterologous prime-boost (HPB) strategy involving DNA followed by PROT (DNA-PROT); and 4) a Control including empty pVAX1 plasmid followed by raw extract of wild-type baculovirus-infected *T. ni* larvae. Hamsters were challenged with *L. infantum* promastigotes and maintained for 20 weeks. While PROT vaccine was not protective, DNA vaccination achieved protection in spleen. Only DNA-PROT vaccination induced significant NO production by macrophages, accompanied by a significant parasitological protection in spleen and blood. Thus, the DNA-PROT strategy elicits strong immune responses and high parasitological protection in the clinical model of VL, better than its corresponding naked DNA or protein versions. Furthermore, we show that naked DNA coupled with raw recombinant proteins produced in insect larvae biofactories –the cheapest way of producing DNA-PROT vaccines– is a practical and cost-effective way for potential “off the shelf” supplying vaccines at very low prices for the protection against leishmaniases, and possibly against other parasitic diseases affecting the poorest of the poor.

## Introduction

Parasitic diseases are major causes of human disease and misery. They plague billions of people among the poorest, killing millions annually, and additionally causing millions of disability-adjusted life years lost *(*DALY). New strategies are urgently needed for diagnosis, treatment and prevention of these pathogens, however the difficulty of cultivating parasites in vitro, their complex organization and life cycle, coupled with its antigenic variability are hampering the results [1].

Leishmaniases can serve as model for other parasitic diseases. Leishmaniases are diseases caused by species of the kinetoplastid parasite *Leishmania* spp. and transmitted by hematophagous sandflies. Leishmaniases represent a major, although grossly underestimated, health problem: over 350 million people are at risk, with a worldwide prevalence of more than12 million cases. The clinical presentation is dependent upon both the parasite species and the host’s immune response. Visceral leishmaniasis (VL) is the most severe form of the disease with deadly epidemics that periodically flare up but go mostly unnoticed. VL has an estimated annual incidence of 500 000, a 90% mortality rate if left untreated, and accounting for around 70 000 deaths per year, thus ranking second only to malaria for mortality and fourth for morbidity amongst tropical parasitic diseases [2], [3], [4]. *Leishmania donovani* causes anthroponotic VL, whereas *L. infantum* (syn. *L. chagasi*) is the causative agent of the zoonotic form, with the domestic dog as its main reservoir.

The control of the disease, as it is the case for most parasitic diseases, is almost confined to chemotherapy, but there are limited number of drugs available, requiring long periods of administration, inducing serious side effects, prone to resistance development, and not affordable for the poor [5]. Currently, it is widely accepted that the concomitant immunity associated to resistance to both human VL and canine leishmaniasis (CanL) is associated to a skewed Th1-like immune response with an antigen-specific CD4^+^ T-cell population producing IFN-γ, which in turns activates macrophages to produce NO, the underlying molecule responsible for the intracellular amastigote death [6], [7]. In fact, the European Medicines Agency has awarded marketing authorization for the prophylaxis and early treatment of CanL to domperidone (Leisguard®, Laboratorios del Dr. Esteve, Barcelona, Spain) [8], an antidopaminergic drug that can act as a proinflammatory cytokine able to skew immune response towards a Th1-like immune response, with cell-mediated immunity (CMI) inducing natural killer cell induction and macrophage activation. Thus, vaccines have been proposed as major cost-effective tools [9], [10] and have been established as a high priority by the World Health Organization (resolution EB118.R3, Geneva 05/07). However, no vaccine against human VL has been marketed to date. Two vaccines have granted marketing authorization against CanL in Brasil –Leishmune® (Pfizer Saúde Animal, São Paulo, Brasil) [11] and Leish-Tec® (Hertape Calier Saúde Animal, Juatuba, Brasil) [12]– and another one has just get a European registration –CaniLeish® Virbac, Carros, France). However, the efficacy of these vaccines remains controversial, particularly when compared with those against viral and bacterial infections.

Several vaccination strategies against VL have been assayed [13], [14]. Killed vaccines have generally been immunogenic yet ineffective. Recombinant protein-based vaccines have achieved moderate protection in mice and dogs, but they often need to be formulated with an adjuvant, which complicates the process for getting marketing authorization, particularly in human medicine. Naked DNA vaccines have also been tested reaching different degrees of protection in rodents, but they have often proved inadequate in providing protection in non-murine models [15]. The only two naked DNA vaccines assayed against *L. infantum* in dogs were not protective [16], [17]. In order to enhance inmunogenicity, DNA vaccines can be improved by the heterologous prime-boost (HPB) strategy, which potentially allows for higher degrees of immunity and protection. This strategy selectively amplifies memory T cells specific for the vaccine antigen [18]. Prime-boost assays carried out against *Leishmania* with the antigen LACK achieved protection in mice [19], but was only moderate in dogs [16], [20], [21]. In contrast, prime-boost vaccination using CPA and CPB antigens was highly protective in both mice [22] and dogs [23]. A cocktail of different evolutionary conserved antigens would probably provide the best protection against the parasite, as suggested elsewhere [24].

We have shown that insect-derived recombinant proteins produced in insect larvae used as living biofactories, even in its raw form, are useful for diagnosis [25], [26], [27], [28], [29], [30], [31], including that of leishmaniasis [32], [33], and vaccination [34], [35], [36], [37].

In this study, we used four evolutionarily conserved *L. infantum* antigens –KMPII (kinetoplastid membrane protein-11, formerly known as KMP-11), TRYP (tryparedoxin peroxidase, previously known as TSA), LACK (*L. infantum* homologue of receptors for activated C kinase), and PAPLE22 (potentially aggravating protein of *L. infantum*)–, formerly shown to be potent B-cell and T-cell immunogens in *L. infantum*-infected dogs [32], to simultaneously compare the protection induced using three vaccination strategies: naked plasmid DNA (DNA), raw insect-derived recombinant protein (PROT), and HPB with naked plasmid DNA followed by raw insect-derived recombinant protein (DNA-PROT). There are several mouse-based models for modeling the immunity against *Leishmania*, however vaccine studies against VL are hampered by the lack of bio-models that accurately reflect the human disease, being the best the golden hamster when experimental infection is used, and the dog for natural infections [38]. Thus, we used the golden hamster model of *L. infantum* infection, known to be the best mimicking the outcome of this disease, characterized by parasite visceralization, splenomegaly, cachexia, and progressive hypergammaglobulinemia [39], [40]. Susceptibility of the hamster to VL is attributed to impaired macrophage effector function, as iNOS transcription is relatively unresponsive to IFN-γ, a finding also reported in humans [41], [42]. Potential drawbacks of the model are that it uses outbred animals and suffers from lack of immunological reagents and assays needed for the dissection of immune responses [4], [43].

## Results

### Safety

The first dose of protein vaccine was well tolerated, but the second one induced transitory pruritus, which resolved within a few hours. This occurred for both wild-type (Ni) and recombinant protein extracts. DNA vaccination was well tolerated, and adverse reactions were not noticed.

### Immunogenicity

Specific seroreactivity to crude total *L. infantum* antigen (CTLA) was not detected before challenge in any vaccinated hamster. Seroreactivity against recombinant antigens could not be evaluated in groups immunized with *T. ni* protein extracts –PROT, DNA-PROT, and control (C)– due to the non-specific responses induced against larva antigens. In the DNA group, specific responses were not detected against the recombinant antigens KMPII, TRYP, or PAPLE22, with only one hamster showing specific anti-rLACK antibodies.

**Figure 1:**
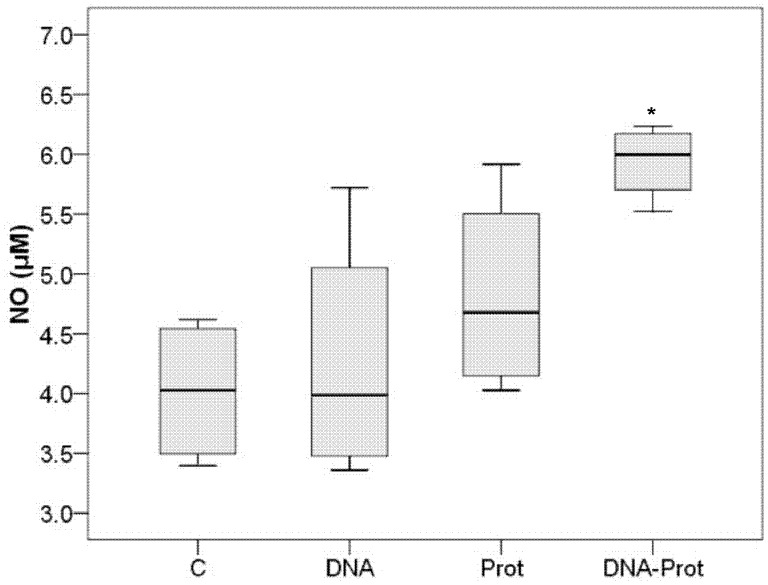
Box plot displaying minimum, first quartile, second quartile (median), third quartile, maximum, and outliers of the production of NO achieved using different vaccination strategies. Production of NO by *Leishmania infantum*–infected macrophages obtained from hamsters vaccinated with KMPII, TRYP, LACK, and PAPLE22 in the form of raw extracts of *Trichoplusia ni* larvae producing recombinant proteins (PROT; n = 4), naked plasmid (DNA; n = 4), both vaccines combined in a prime-boost strategy (DNA-PROT; n = 4), and in control hamsters (C; n = 4). Each sample was analyzed in triplicate. (*P = 0.029).

*In vitro* infected macrophages from DNA-PROT vaccinated hamsters produced a significantly higher quantity of NO than those from the C group (Mann-Whitney *U*; *P = *0.029), while no differences were observed between DNA or PROT groups and the C group (Figure 1). No significant differences were found between the number of amastigotes infecting macrophages from the vaccinated groups and the C group at 72 h post-*in vitro* infection (Mann-Whitney *U*; *P* > 0.050 for all comparisons).

### Parasitological Protection

**Figure 2:**
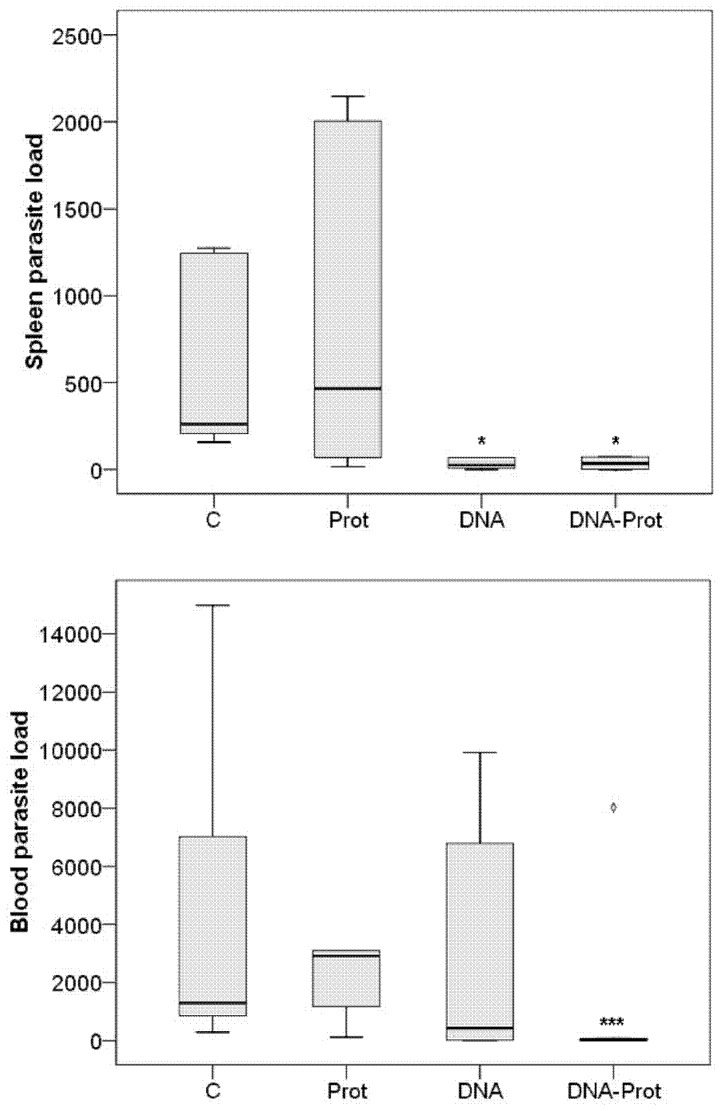
Parasite load achieved using different vaccination strategies. Parasite load in spleen (relative, fold more DNA copies than the calibrator) and parasite load in blood (parasites/mL) 20 weeks after *Leishmania infantum* challenge in hamsters vaccinated with KMPII, TRYP, LACK, and PAPLE22. C: Control (n = 6); PROT: Raw extracts of *Trichoplusia ni* larvae producing recombinant proteins (n = 6); DNA: Naked plasmid DNA (n = 6); DNA-PROT: Both vaccines combined in a prime-boost strategy (n = 5). Each sample was analyzed in triplicate.

The descriptive statistical analysis (Figure 2) identified an extreme outlier in the blood parasite load from the DNA-PROT group (Grubbs’ test; *P = *0.026 and 3.0× the interquartile range outside the central box [44]), which was excluded in the subsequent inferential analysis. When compared to the C group, PROT vaccine was not protective neither in spleen (Mann-Whitney *U*; *P = *0.818) nor in blood (Mann-Whitney *U*; *P = *0.179). In contrast, DNA vaccine achieved significant parasitological protection in spleen (Mann-Whitney *U*; *P = *0.015), but was not protective in blood (Mann-Whitney *U*; *P = *0.792). Finally, DNA-PROT vaccine induced significant parasitological protection in spleen (Mann-Whitney *U*; *P = *0.030) and highly significant parasitological protection in blood (Mann-Whitney *U*; *P* < 0.001). The results are shown in Figure 2.

One hamster from the DNA group and another one from DNA-PROT group showed absolute sterile protection both in blood and spleen.

### Specific Immune Response Induced Upon *L. infantum* Challenge

**Figure 3:**
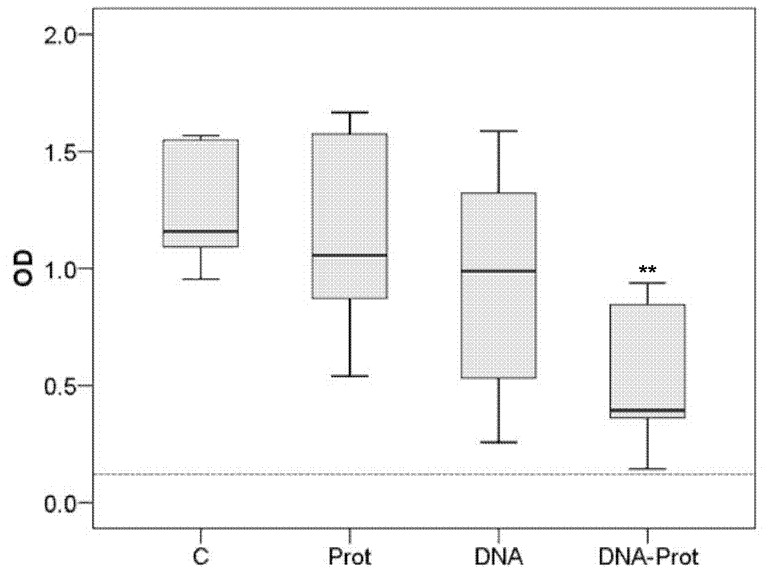
Seroreactivity achieved using different vaccination strategies. Seroreactivity against crude total *Leishmania* antigen (CTLA) 20 weeks after *Leishmania infantum* challenge in hamsters vaccinated with KMPII, TRYP, LACK, and PAPLE22. C: Control; PROT: Raw extracts of *Trichoplusia ni* larvae producing recombinant proteins; DNA: Naked plasmid DNA; DNA-PROT: Both vaccines combined in a prime-boost strategy. The dotted line shows the cut-off value.

Concentration of specific anti-*L. infantum* antibodies in hamsters from the DNA and PROT groups were not significantly different to those from the C group (Mann-Whitney *U*; *P* > 0.050 for both comparisons). Conversely, the DNA-PROT group produced significantly lower anti-CTLA antibody concentrations than the C group (Mann-Whitney *U*; *P = *0.004). The results are shown in Figure 3. The concentration of antibodies against CTLA significantly correlated with the parasite load both in blood (Spearman’s *ρ*; *P* < 0.001) and spleen (Spearman’s *ρ*; *P* < 0.001).

Seroreactivity against recombinant antigens could not be evaluated in the PROT and DNA-PROT groups due to the antibody responses induced against larva proteins. In the DNA group, one hamster showed antibodies against rKMPII, rLACK, and rPAPLE22, and another one developed antibodies against rLACK. Seroreactivity against rTRYP was not detected.

### Histopathology

Findings compatible with leishmaniasis –follicular hyperplasia, mononuclear inflammatory infiltrate in the periportal areas, and hyperplasia of Kupffer cells in the sinusoids– were milder in the DNA-PROT group in relation to the C group. Those hamsters achieving absolute sterile protection, both in blood and spleen, showed no histopathological abnormalities in the spleen or liver. *No histological abnormalities were found elsewhere.*

## Discussion

We used the preclinical golden hamster model of VL for a head-to-head simultaneous comparison of the protective efficacy of three vaccination strategies –based on the antigens KMPII, TRYP, LACK, and PAPLE22– against *L. infantum* infection: raw insect-derived recombinant protein (PROT), DNA, and HPB DNA-PROT.

In spite of the fact that vaccination with recombinant proteins has been described as moderately effective against *L. infantum* infection in rodents [45], [46], the immunization of hamsters with crude rKMPII, rTRYP, rLACK, and rPAPLE22 expressed in *T. ni* larvae did not achieve parasitological protection, and we could not detect specific CMI. Raw protein extracts of larvae include remains of baculovirus and it has been shown to stimulate proinflamatory cytokines involved in the stimulation and maintenance of Th1 cellular immune responses [47], including those against *Leishmania*. This stimulation occurs through the MyD88-TLR9 pathway, being TLR9 the innate receptor for unmethylated CpG motifs –present in the baculoviral DNA–, that are considered pathogen-associated molecular patterns and responsible for the immunostimulatory Type I proproinflamatory response [48]. In this way, the intrinsic immunostimulatory property of baculovirus may alleviate the requirement of traditional adjuvants added to the vaccine formulations [49], [50], [51]. In our case this stimulation appears not to be enough, and the lack of traditional adjuvants might explain the failure to induce a Th1 response [24]. However, few adjuvants have been approved for human vaccines, and they currently appear inadequate to confer protection against other intracellular parasitic diseases [52]. In contrast, other vaccines using raw insect-derived recombinant proteins yielded successful results against viral diseases [53], even in the absence of an adjuvant [34] . This fact probably reflects the strong CMI required for protection against intracellular parasites, or a deleterious effect of some of the vaccine antigens used in our assay. KMPII [54], LACK [55], and PAPLE22 [56] elicit human production of IL-10, an anti-inflammatory cytokine capable of inhibiting synthesis of Th1-like cytokines, which has also been described in infected hamsters [57].

DNA vaccines have proven capable of stimulating antibody production, as well as CD4+ and CD8+ T CMI [58]. In our study, the DNA vaccine induced antibodies against rLACK in one hamster, but antibodies against the other vaccine antigens were not detected, as it has been reported for other DNA candidates [59]. Despite the lack of parasitological protection in blood, the significant parasitological protection in spleen –that achieved sterility in some animals– suggests that some degree of CMI response was induced, although not enough for significant NO production. DNA vaccine could have a deleterious effect on aberrant B-cell proliferation and antibody production associated with disease [39], as it prevented hyperplasia of the lymphoid follicles after challenge.

HPB vaccination using raw insect-derived recombinant proteins after DNA achieved almost full parasitological protection in blood and spleen, reaching sterility in some individuals. This protection was associated with significant production of NO in the macrophages, indicative of effective T-cell mediated anti-*L. infantum* activity. Other studies have described an association between vaccine protection and levels of NO production [60] or iNOS expression [61] in hamster macrophages. Despite the relative unresponsiveness of iNOS transcription to IFNγ in the hamster [42], other cytokines, such as IL-1, TNF, IFNα, or IFNβ might induce NO production and protection [62]. Moreover, a lower parasite-specific humoral response was observed in the DNA-PROT group in relation to the C group after challenge. This phenomenon –also described in hamsters vaccinated with *PAPLE22* DNA [63] and in other protective vaccines against VL in hamsters [61] and dogs [12], [21]– might be due to a redirection of the Th2-like response towards a Th1-like response and/or to the lower antigenic stimulation caused by lower parasite loads. In this sense, our results showed a positive correlation between parasite load in spleen and blood samples and the *L. infantum*-specific humoral response, as previously described in hamsters [40]. The DNA-PROT vaccine also prevented certain histopathological alterations in spleen and liver that have been attributed to leishmaniasis in hamsters and dogs [40], [64].

Attempts to enhance the protective immune responses against experimental murine leishmaniasis have been successful using HPB vaccination strategies based on successive administration of different DNA vector vaccines [65], [66]. A number of antigen candidates have been assayed on the murine model of VL in a HPB approach based on DNA followed by protein produced using traditional cell-culture technology, such as, ORFF [67], CPs [22], and LACK [19]. Moreover, comparative vaccine potential of DNA, protein, and HPB vaccination was evaluated against leishmaniasis in the murine model [68], [69]. Unfortunately, experimental models often lack consistency with clinical settings [70], [71], thus being the major challenge the translation of results from animal bio-models to human disease and the transition from the laboratory to the field [72]. Because of the many analytical tools available, the murine model of VL is an excellent one to dissect the immunological bases but, when research is focused on the disease, it is a very poor model because in most situations they clear infection from both spleen and liver providing very little medical translational information. Conversely, the hamster model of VL has very little tools for studying immunity being an incomplete model for immunological studies, but it reproduces with high fidelity the clinical human disease [39], [73]. Thus, when studying the disease, not their immunological bases –that very often do not translate directly into clinical disease–, the hamster model is very robust and, by far, better and preferable over the murine one, because of the added value of the high medical translational information obtained.

To the authors’ knowledge, this is the first head-to-head simultaneous comparison between naked DNA, raw insect-derived recombinant protein, and HPB strategies performed using a reliable and robust clinical model of VL. Our results show that the HPB strategy with naked DNA and raw insect-derived recombinant proteins –rKMPII, rTRYP, rLACK, and rPAPLE22– elicitis a strong CMI response and parasitological protection against *L. infantum*, better than that obtained with either the naked DNA or protein vaccines used alone. Last but not least, our results demonstrate that using naked DNA –the cheapest way of obtaining DNA vaccines– coupled with raw recombinant proteins produced in insect larvae biofactories –capable of producing large quantities of recombinant protein per biomass unit at low production costs and easy to scale-up [36]– is a practical and cost-effective way for “off the shelf” supplying vaccine doses at a very low price for the protection against leishmaniases, and possibly against other parasitic diseases affecting the poorest of the poor.

## Materials and Methods

### Ethics Statement

The experimental design and analyses have been planned to ensure cost-benefit in the number of animals used and according to the Three Rs [74], [75]. The experimental procedures involved in this research have been approved by the Ethics Committee on Animal and Human Research of the Universitat Autònoma de Barcelona (CEEAH 662), in accordance with the ethical and legal requirements (5/1995/Generalitat de Catalunya, 214/1997/Generalitat de Catalunya, Real Decreto 1201/2005, 86/609/CEE, 91/628/CEE and 92/65/CEE) concerning the use of animals in research, and registered by the Department de Medi Ambient of the Generalitat de Catalunya (DMAH 3922).

### Gene Cloning and DNA Vaccine Construction

Gene cloning was performed as previously described [17]. The primers used to amplify the coding sequences of the *KMPII*, *TRYP*, and *LACK* genes have been described elsewhere [17] and the coding region of *PAPLE22* (GenBank accession number AF123892) was amplified using forward 5′-GGCCACTTCTCTCTTCTCCA-3′ and reverse 5′-CTTGCCACATACACCAATCG-3′ primers. The coding sequences of each gene were cloned separately into the pVAX1™ vector (Invitrogen™, Carlsbad, CA, USA). Large-scale plasmid preparations were performed using an EndoFree® Plasmid Giga Kit (Qiagen®, Valencia, CA, USA) according to the manufacturer’s instructions.

### Recombinant Proteins and Protein Vaccine Production

Recombinant proteins were obtained in baculovirus-infected *Trichoplusia ni* larvae as described elsewhere [32], [33]. Wild-type baculovirus was used to obtain the control raw protein extract (Ni antigen) for vaccination and ELISA. The concentrations of specific recombinant proteins in the raw *T. ni* extracts were 1% for recombinant KMPII protein (rKMPII), 0.5% for rTRYP, 2% for rPAPLE22, and 5% for rLACK. Antigenic characterization of the recombinant proteins has been explained elsewhere [32], [33].

### Experimental Design, Animals and Immunizations

Forty-four ten-week-old male golden hamsters (*Mesocricetus auratus*, outbread strain Han:AURA) purchased from a commercial source (Centre d’Elevage René Janvier, Le Genest-Saint-Isle, France) were used for this study. The animals, housed in micro-filter cages at animal biosafety level 2 facilities, were kept and handled by a veterinarian.

Forty hamsters were randomly assigned to four equally sized experimental groups: protein vaccine (PROT), naked DNA vaccine (DNA), prime-boost vaccine (DNA-PROT), and control (C). One hamster from the DNA-PROT group died during the acclimatizing period and was not substituted. All groups were handled in parallel so that infection inoculum was the same and administered at the same time, as it was the case for vaccinations and euthanasia. Samples from four additional hamsters that were neither vaccinated nor infected (NN) were used as controls for the different techniques.

The PROT group received 2 intraperitoneal doses of a mixture of raw larvae extracts containing 5 µg of each recombinant protein in 200 µL of sterile saline solution. The DNA group received 3 intramuscular doses of 100 µg of each plasmid construction in 100 µL of sterile saline solution (50 µL into each *tibialis cranialis* muscle). The DNA-PROT group received 3 doses of DNA vaccine, followed by 2 doses of protein vaccine using the same protocols as for the DNA group and the PROT group. Finally, the C group received 3 doses of pVAX1**™** without insert (400 µg) in 100 µL of sterile saline solution, followed by 2 doses of Ni antigen, using the equivalent amount of total protein as in the DNA-PROT group. The interval between immunizations was 2 weeks. The safety of the vaccine prototypes was assessed at 1 h, 4 h, 24 h, 48 h, and 72 h after each vaccination by close observation and clinical examination of the animals. No adjuvants were used in order to not to interfere with the actual protective response of the different vaccination strategies and to allow comparisons.

Valid surrogate markers of protection against human leishmaniasis and in clinical bio-models are still lacking. The most widely used measure of vaccine potential has been to monitor anti-*Leishmania* immune responses, either humoral or cellular, over time but nucleic acid detection is an established method for detection of *Leishmania* parasite in blood and other tissues [38]. As explained by WHO guidelines [76], confirmation of infection by very sensitive methods such as PCR or culture represent a very early end-point of the development of the disease [77]. Thus, primary outcome measures were differences between groups in parasite load and in NO production, the main functional effector responsible for killing *Leishmania* and for controlling the infection [78]. Secondary outcomes were differences in specific humoral immune response, parasite killing, and histopathology.

### Parasites and Challenge

Two weeks after the last immunization, four hamsters from each vaccine group were euthanized to evaluate the immunogenicity of each vaccination schedule. The remaining hamsters were challenged with an intraperitoneal injection of 1 × 10^7^ stationary promastigotes of *L. infantum* (strain MCAN/ES/92/BCN-83/MON-1), clinically evaluated once a week for the presence of signs compatible with leishmaniasis, and euthanized 20 weeks after experimental infection. Parasites –kindly provided by Dr. M. Gállego, Grup de Parasitologia Clínica, Universitat de Barcelona, Spain– were obtained from a naturally infected dog that had not received any treatment and were passaged through hamsters in order to retain their full virulence.

### Necropsy and Tissue Sampling

Hamsters were anesthetized by a 100 µL intramuscular injection containing 42.5 mg·mL^−1^ ketamine, 9.5 mg·mL^−1^ xylazine, and 1.5 mg·mL^−1^ acepromazine. Then, a 5****mL sample of intracardiac blood was collected and the animals were euthanized using CO_2_.

Peritoneal macrophages were collected by intraperitoneal injection of cold Gibco Dulbecco’s Modified Eagle Medium (DMEM) (Invitrogen™, Carlsbad, CA, USA) supplemented with 5% (v/v) FCS and 1% (w/v) EDTA, only from hamsters euthanized after vaccination.

A sample of spleen tissue was collected and snap-frozen in liquid nitrogen. Spleen size was roughly estimated at necropsy as length × width (cm^2^). Normal spleen size range was estimated on the basis of the results from hamsters in the NN group and calculated as the mean area ± 3 SD. Spleens larger than 2.7 cm^2^ were considered to be abnormally enlarged.

Samples of spleen, liver, and kidney were collected and fixed in buffered 10% formaldehyde solution.

### Crude Total Antigen and Recombinant Antigen-based ELISA

The specific humoral immune response against CTLA and recombinant *L. infantum* antigens was measured by ELISA as previously described [32], [79].

For recombinant antigen-based ELISAs, each serum sample was tested against each raw protein larva extracts containing rKMPII, rTRYP, rLACK, rPAPLE22, and their corresponding control Ni antigen prepared at the same concentration in the same plate. For CTLA-ELISA, plates were coated with 2 µg of CTLA per well. Working dilutions for sera were 1∶10 (recombinant antigen-based ELISAs) or 1∶400 (CTLA-ELISA). Anti-hamster IgG antibody (Sigma-Aldrich®, St. Louis, MO, USA) diluted 1∶1000 was used as a secondary antibody. A known positive serum used as calibrator was included in all plates, and plates with interassay variations ≥ 10% were ruled out. The cut-off values for both types of ELISA were established on the basis of optical densities obtained from the NN group and calculated as the mean + 3 SD, resulting in 0.122 for CTLA, 0.266 for rKMPII, 0.050 for rTRYP, 0.284 for rLACK, and 0.376 for rPAPLE22.

ELISA results were expressed as optical densities (OD). For those using recombinant antigens, absorbances were corrected by subtracting the absorbance achieved by the serum on the control antigen Ni extract from that achieved by the protein larva raw extract containing specific recombinant antigen.

### Quantification of NO Production and Parasite Killing by Macrophages Infected *in vitro*

Peritoneal macrophages were harvested at 2 × 10^5^ per well in a 16-well glass Lab-Tek™-Chamber Slide™ System (Nalge Nunc International, Rochester, NY, USA). *In vitro* infection was performed with stationary-phase promastigotes of *L. infantum* (MCAN/ES/92/BCN-83/MON-1) at a parasite:macrophage ratio of 7∶1 for 4 h at 37°C in 5% CO_2_. Non-internalized parasites were removed by gentle washing with pre-warmed PBS, and infected macrophages were then cultured in Gibco DMEM without Phenol Red (Invitrogen™, Carlsbad, CA, USA) supplemented with 5% (v/v) FCS for 72 h. Supernatants were then collected and stored at −80°C, and slides were Giemsa-stained.

Culture supernatants were analyzed for their NO concentrations using the Griess reaction by measuring combined oxidation products of NO (NO_2_^−^ and NO_3_^−^) after reduction with nitrate reductase in a Colorimetric Assay (Cayman Chemical Company, Ann Arbor, MI, USA). The number of amastigotes per 100 macrophages was determined by microscopic examination of Giemsa-stained slides.

### Real-time PCR Amplification of *L. infantum* DNA from Spleen and Blood Samples

*L. infantum* DNA was specifically detected and quantified as described elsewhere using the Taqman® real-time PCR (qPCR) (Applied Biosystems™, Foster City, CA, USA) targeting conserved DNA regions of the kinetoplast minicircle DNA, and the eukaryotic 18S RNA Pre-Developed TaqMan Assay Reagents (Applied Biosystems, Foster City, CA, USA) as internal reference gene. For spleen, relative quantification was performed by the 2^−ΔΔCt^ method [80] using as calibrator the sample showing the lowest ΔCt and results expressed as x-fold more DNA copies than this calibrator sample [81]. For blood, quantification was performed by the 2^−ΔΔCt^ method [80] using as calibrators spiked samples with a known number of parasites/well, thus allowing determining the number of parasites in any PCR sample, independently of the amount of DNA added or the presence of inhibitors, and results expressed as parasites/mL [82].

### Histopathology

Samples of liver, spleen and kidney were embedded in paraffin, and sections were stained by hematoxylin and eosin for histopathological study with high-power light microscopy at ×400.

### Statistical Analysis

Two-tailed non-parametric techniques were used throughout the study. Statistical significance for *a posteriori* comparisons was corrected for multiple simultaneous comparisons employing a rough false discovery rate [*83*]. All analyses were carried out using SPSS v.14.0 (SPSS Inc, Chicago, IL, USA).
